# Associations of Suboptimal Growth with All-Cause and Cause-Specific Mortality in Children under Five Years: A Pooled Analysis of Ten Prospective Studies

**DOI:** 10.1371/journal.pone.0064636

**Published:** 2013-05-29

**Authors:** Ibironke Olofin, Christine M. McDonald, Majid Ezzati, Seth Flaxman, Robert E. Black, Wafaie W. Fawzi, Laura E. Caulfield, Goodarz Danaei, for the Nutrition Impact Model Study (anthropometry cohort pooling)

**Affiliations:** 1 Department of Epidemiology, Harvard School of Public Health, Boston, Massachusetts, United States of America; 2 Department of Nutrition, Harvard School of Public Health, Boston, Massachusetts, United States of America; 3 Department of Global Health and Population, Harvard School of Public Health, Boston, Massachusetts, United States of America; 4 School of Computer Science and Heinz College, Carnegie Mellon University, Pittsburgh, Pennsylvania, United States of America; 5 Department of International Health, Johns Hopkins Bloomberg School of Public Health, Baltimore, Maryland, United States of America; 6 MRC-HPA Center for Environmental Health, Department of Epidemiology and Biostatistics, School of Public Health, Imperial College London, London, United Kingdom; Indiana University, United States of America

## Abstract

**Background:**

Child undernutrition affects millions of children globally. We investigated associations between suboptimal growth and mortality by pooling large studies.

**Methods:**

Pooled analysis involving children 1 week to 59 months old in 10 prospective studies in Africa, Asia and South America. Utilizing most recent measurements, we calculated weight-for-age, height/length-for-age and weight-for-height/length Z scores, applying 2006 WHO Standards and the 1977 NCHS/WHO Reference. We estimated all-cause and cause-specific mortality hazard ratios (HR) using proportional hazards models comparing children with mild (−2≤Z<−1), moderate (−3≤Z<−2), or severe (Z<−3) anthropometric deficits with the reference category (Z≥−1).

**Results:**

53 809 children were eligible for this re-analysis and contributed a total of 55 359 person-years, during which 1315 deaths were observed. All degrees of underweight, stunting and wasting were associated with significantly higher mortality. The strength of association increased monotonically as Z scores decreased. Pooled mortality HR was 1.52 (95% Confidence Interval 1.28, 1.81) for mild underweight; 2.63 (2.20, 3.14) for moderate underweight; and 9.40 (8.02, 11.03) for severe underweight. Wasting was a stronger determinant of mortality than stunting or underweight. Mortality HR for severe wasting was 11.63 (9.84, 13.76) compared with 5.48 (4.62, 6.50) for severe stunting. Using older NCHS standards resulted in larger HRs compared with WHO standards. In cause-specific analyses, all degrees of anthropometric deficits increased the hazards of dying from respiratory tract infections and diarrheal diseases. The study had insufficient power to precisely estimate effects of undernutrition on malaria mortality.

**Conclusions:**

All degrees of anthropometric deficits are associated with increased risk of under-five mortality using the 2006 WHO Standards. Even mild deficits substantially increase mortality, especially from infectious diseases.

## Introduction

Restricted growth as a result of inadequate nutrition and infections is an important cause of morbidity and mortality in infants and children worldwide [1]–[3]. It has been estimated that 30% of children under five (170 million) in the world are moderately or severely stunted, and 19% (110 million) are moderately or severely underweight [4]. In addition to these, 144 million have mild stunting and 148 million mild underweight [4]. Several prospective studies have shown associations of undernutrition with increased risk of various disease outcomes, and reduced survival, in children [5]–[14].

A 1994 meta-analysis of 8 studies in 6 countries [15] reported mortality rate ratios ranging from 2.5 to 8.4 for mild and moderate underweight children, relative to a ‘normal’ weight-for-age. The 2004 World Health Organization (WHO) Comparative Risk Assessment (CRA) [16] Study was the first global pooling project that analyzed the association of weight-for-age Z score (WAZ) with mortality in four categories: WAZ <−3, WAZ −3 to <−2, WAZ −2 to <−1 and WAZ≥−1. The pooled analysis was updated in the 2008 Lancet Series on Maternal and Child Undernutrition [1]. This analysis further investigated height/length-for-age Z score (HAZ, a measure of stunting) and weight-for-length/height Z score (WHZ, a measure of wasting) as other anthropometric measurements. Virtually all mortality effect estimates in the 2008 Lancet Series were smaller than those reported in the CRA Study, by as much as 83%. At the extreme, the CRA Study found that all levels of underweight significantly increased malaria mortality rates with rate ratios ranging from 2.1 (1.48, 3.02) for mild underweight to 9.5 (3.25, 27.66) for severe underweight, while the 2008 Lancet Series results found no significant association of underweight with malaria mortality, with odds ratios ranging from 0.8 (0.2, 3.2) to 1.6 (1.0, 2.7) for different degrees of underweight.

The two studies differed in several aspects which makes it difficult to assess why the associations changed and what effect sizes should be used for assessing the health consequences of suboptimal growth. First, the cohorts used in the two pooling analyses differed, although 7 common cohorts were included in both analyses. Second, the CRA Study used the 1977 U.S. National Center for Health Statistics/WHO International Growth Reference (NCHS/WHO 1977) [17] to define WAZ, while the 2008 Lancet Series defined WAZ, HAZ and WHZ using the 2006 World Health Organization (WHO) Child Growth Standards (WHO 2006) [18]. Third, the CRA Study estimated mortality rate ratios while the 2008 Lancet Series estimated mortality odds ratios. Fourth, the CRA Study extracted and pooled results of existing studies for computing summary effect estimates whereas the Lancet Series ran Generalized Linear Mixed models using individual-level data. Fifth, the CRA Study could not adjust for confounding because the authors did not have access to individual-level data; the 2008 Lancet Series applied a 15% attenuation to the estimated odds ratios to adjust for confounding by some socioeconomic factors [1].

The renewed policy and programmatic attention to child nutrition and growth means that there is a need for definitive estimates of the association between children’s anthropometric status and total and cause-specific mortality. Examples of such policies and programs include the United Nations (UN) Secretary-General’s “Every Woman Every Child” [19] global movement with the accompanying “Global Strategy for Women’s and Children’s Health” [19], [20] and the more recent United Nations Children’s Fund (UNICEF) “Committing to Child Survival: A Promise Renewed” [21] initiative for ending preventable child deaths, as well as the “Scaling Up Nutrition” movement [22]. It is also important for clinicians, program designers and policy makers to understand how the change in growth standards has affected the mortality effect estimates among children classified as stunted, wasted, or underweight, using comparable data and methods. Finally, the availability of data on the full distributions of anthropometric indicators [4] makes it desirable to assess the association with mortality in narrower Z score bands.

To address these issues, we collated and analyzed data from 10 large prospective studies in low- and middle-income countries. We used these cohorts to investigate the association between different indicators of suboptimal growth and mortality in children 1 week to 59 months old, using both the more recent WHO 2006 standards and the older NCHS/WHO 1977 reference and employing identical methods. We also assessed how effect sizes are affected by adjustment for potential confounding and used narrower Z score bands to estimate a more detailed dose-response relationship between suboptimal growth and mortality.

## Methods

### Ethics Statement

Pooled analysis of anonymized data was assessed as exempt by the Harvard School of Public Health’s Institutional Review Board.

### Study Selection

The principal investigators of all studies that were included in the analysis of child undernutrition and mortality in the CRA Study [16] and the 2008 Lancet Series [1] were contacted to request individual-level data. We included prospective cohort studies or randomized trials that measured height and weight and assessed vital status of children during follow-up. We successfully obtained data from nine of the ten studies included in the CRA Study [23]–[30] and seven of the eight studies in the 2008 Lancet Series [23]–[25], [27], [29], [30] (both earlier projects included studies in Pakistan which are not part of the current analysis). Anonymized data from ten studies in Bangladesh [25], Ghana [23], Guinea-Bissau [29], India [23], Indonesia [28], Nepal [30], Peru [23], the Philippines [24], Senegal [27], and Sudan [26] were included in this analysis. Five of the selected studies were randomized controlled trials of vitamin A supplementation [23], [26], [30].

### Eligibility and Anthropometric Assessment

Eligible participants were children who were one week to 59 months old and had at least one recorded visit in which weight or height (or length) was measured. We computed WAZ, HAZ and WHZ using the WHO growth standards of 2006 [18] as well as the NCHS/WHO 1977 reference [17]. We truncated extreme length/height values (defined as <45 cm or >110 cm for children <2 years of age, and <65 cm or >120 cm for children between 2 and 5 years of age) and set them to the limits of the defined plausible range to prevent undue influence of extreme values. In the main analysis, categories of WAZ, HAZ and WHZ were defined as mild (−2≤Z<−1), moderate (−3≤Z<−2), or severe (Z<−3) anthropometric deficits with the reference category being no deficit (Z≥−1). For analyses involving finer categories, we used decrements of 0.5 Z scores down to <−4, with the same reference category (i.e., Z≥−1).

Weight and height/length were measured multiple times during follow-up in all cohorts. Each child’s Z scores and related anthropometric status category at the most recent measurement were carried forward until the next visit. We used the most updated anthropometric status at each time point. The median interval between the last anthropometric measurements and death was 11 weeks and the interquartile range was 4 to 16 weeks. Each eligible child contributed person-time until age 59 months, death, or the administrative end of follow-up in the child’s particular cohort, whichever occurred first. The 5-year age limit was used because the WHO 2006 standards were provided for children up to 60 months old [18].

### Outcome Definition

Our primary outcome was death from any cause. The ascertainment procedures of vital status and causes of death for included studies have been reported elsewhere [23]–[30]. Briefly, in all studies vital status was ascertained at regular study visits. Causes of death were available for all cohorts except the study from Indonesia [28]. The other nine studies ascertained cause of death using verbal autopsy methods; four studies also used hospital records when available [23], [29]. In cause-specific analyses, we classified deaths as those due to diarrheal diseases, respiratory tract infections, measles, malaria, and ‘other infectious diseases’ (septicemia, unspecified febrile illness, tuberculosis, meningitis, hepatitis or cellulitis).

### Statistical Analyses

All analyses were conducted separately for WAZ, HAZ, and WHZ. We pooled individual-level data from cohorts and estimated mortality hazard ratios (HR) for children in each anthropometric status category relative to those with Z≥−1, using the counting process formulation of Cox proportional hazards regression models [31]. Cox regression uses the partial likelihood method to estimate hazard ratios, removing the need to specify baseline hazard functions. It allows for use of censored data, i.e. when study participants' event times are unknown for reasons including loss to follow-up or study termination. Hazard ratios are valid when censoring is non-informative. Cox regression also allows for stratification of models to adjust for variables for which the proportional hazards assumption does not hold [32]. We used child’s age (in weeks) as the time scale and stratified models on cohort to allow separate baseline hazards. We further adjusted for child’s sex and the assigned treatment in the randomized trials in our minimally adjusted models. Some additional covariates were available in 6 cohorts (e.g., household assets, mother’s education, household water source, and sanitation. See Table S1) but the available covariates differed across cohorts. Therefore, the maximally-adjusted HRs were estimated separately for each of the 6 cohorts and the HRs were pooled using a random effects meta-analysis using the method of DerSimonian and Laird [33]. We repeated the minimally adjusted analysis in these 6 cohorts using the meta-analysis approach to provide a set of consistent results and evaluated the magnitude of confounding by the additional covariates.

### Sensitivity Analyses

Childhood is a period of relatively rapid growth, and changes in nutrition or disease episodes can change a child’s Z score within weeks/months depending on the measure. While the included studies had measured anthropometric status in relatively short intervals, Z scores from the previous visit may quickly become outdated, leading to measurement error in exposure. To assess the sensitivity of our results to how long WAZ, HAZ and WHZ values were carried forward, we conducted a sensitivity analysis in which we carried the last observed anthropometric measurement only up to 4 months (after which the person-time from the child was censored). In this sensitivity analysis, the median time interval between anthropometric measurements and death was 8 weeks (interquartile range 3 to 12 weeks).

We also conducted a sensitivity analysis on cause of death classification. Specifically, 39 deaths in two studies were reported to be due to both respiratory tract infections and diarrheal diseases. In the main analysis we categorized these deaths under both analyses but in a sensitivity analysis, we alternatively considered these deaths as due to one or the other cause, but not both.

All analyses were performed using SAS version 9.3 (SAS Institute Inc. Cary NC USA) and R version 2.13.0 (2011-04-13).

## Results

Characteristics of the selected cohorts are presented in Table 1. The cohorts included 53 809 children aged 1 week to 59 months old who participated in 10 studies in Asia, Africa and South America. Study recruitment periods ranged from 1977 (Indonesia) to 1997 (Ghana). Participants contributed a total of 55 359 person-years, during which 1315 deaths occurred. In the pooled sample, 63% of children were underweight (WAZ<−1), 67% were stunted (HAZ<−1), and 34% were wasted (WHZ<−1) at baseline. The prevalence proportions of severe baseline underweight, stunting and wasting were 12%, 20% and 3%, respectively. Table 2 presents the age distribution of children at study entry and exit. Approximately one third of children were 0–5 months old at entry; a fifth of the 1315 deaths also occurred in this age group. The 54–59 month group had the fewest deaths.

**Table 1:** Characteristics of included studies.

Author, year	Country	Study design	Study recruitment dates	Age range at recruitment [months]	No. of participants recruited	No. of eligibleParticipants(current analysis)	Proportion female (%)	No. of deaths(current analysis)	Median (total) follow-up for current analysis [years, (person years)]
**Arifeen, 2001 ** [**25**]	Bangladesh	Prospective cohort	1993–1995	0–0.4	1677	1581	49.2	119	1.0 (1240)
**WHO/CHD, 1998 ** [**23**]	Ghana	RCT^a^	1995–1997	0.5–5	2882	2869	50.9	61	0.8 (1902)
**Mølbak, 1992 ** [**29**]	Guinea-Bissau	Prospective cohort	1987–1990	0–50	1165	1145	49	114	1.2 (1267)
**WHO/CHD, 1998 ** [**23**]	India	RCT^a^	1995–1996	1–5	3981	3926	48.3	88	0.9 (3074)
**Katz, 1989 ** [**28**]	Indonesia	Prospective cohort	1977	0–71	4696	3899	48.4	156	1.0 (4488)
**West, 1991 ** [**30**]	Nepal	RCT^a^	1989	0–60	6617	6418	48.4	137	1.6 (8051)
**WHO/CHD, 1998 ** [**23**]	Peru	RCT^a^	1995–1996	1–6	2437	2393	49.2	15	0.8 (1726)
**Adair, 1993 ** [**24**]	Philippines	Prospective cohort	1982–1983	0–3	3080	2948	47.1	110	1.8 (4924)
**Garenne, 2000 ** [**27**]	Senegal	Prospective cohort	1983	0–61	5781	5750	49.5	354	0.9 (4589)
**Fawzi, 1997 ** [**26**]	Sudan	RCT^a^	1988	0–89	29 615	22 880	48.9	161	1.5 (24 098)

a Randomized trial of vitamin A supplementation.

**Table 2:** Age distribution of children at study entry and exit.

	Study entry	Study exit	
Age (months)	No. at baseline (% of all children)	No. censored^a^ (% of all children)	No. of deaths (% of all children)
**0–5**	18480 (34.34)	2704 (5.03)	292 (0.54)
**6–11**	3563 (6.62)	9416 (17.50)	232 (0.43)
**12–17**	4250 (7.90)	2753 (5.12)	191 (0.35)
**18–23**	4282 (7.96)	4076 (7.57)	182 (0.34)
**24–29**	3954 (7.35)	3844 (7.14)	172 (0.32)
**30–35**	4226 (7.85)	4144 (7.70)	100 (0.19)
**36–41**	4014 (7.46)	4230 (7.86)	65 (0.12)
**42–47**	3899 (7.25)	3987 (7.41)	40 (0.07)
**48–53**	3818 (7.10)	4115 (7.65)	27 (0.05)
**54–59**	3323 (6.18)	13225 (24.58)	14 (0.03)
**Total**	53809 (100)	52494 (97.56)	1315 (2.44)

a Number censored includes children who were administratively censored and those lost to follow-up.

### Undernutrition and All-cause Mortality

Even mild anthropometric deficit was associated with significantly higher hazard of dying in childhood, with the strength of association increasing as Z scores decreased (Table 3). For example, using the WHO 2006 standards, severely underweight children (WAZ<−3) died at a rate 9.40 times higher (95% Confidence Interval 8.02, 11.03) than children with WAZ of −1 or greater. Mortality hazards for children with mild and moderate underweight were also significantly higher than those with WAZ≥−1, with HRs of 1.52 (1.28, 1.81) and 2.63 (2.20, 3.14), respectively. Wasting was a stronger risk factor for mortality as compared with underweight or stunting. Children with severe wasting had 11.63 fold higher mortality rates (9.84, 13.76) than children with WHZ≥−1 compared with 5.48 (4.62, 6.50) for severe stunting. Similarly, mortality HRs for moderate and mild wasting were, respectively, 48% and 11% larger than HRs for moderate and mild stunting (Table 3). There was no significant heterogeneity in hazard ratios across studies (see Table S2).

**Table 3:** Minimally adjusted^a^ hazard ratios (HR) using WHO 2006 standards and NCHS/WHO 1977 reference.

	WHO 2006		NCHS/WHO 1977	
	No. of deaths	HR (95% CI)	No. of deaths	HR (95% CI)
**Weight-for-Age Z score**				
**<**−**3**	489	9.40 (8.02, 11.03)	411	12.75 (10.48, 15.50)
−**3 to<**−**2**	284	2.63 (2.20, 3.14)	366	3.84 (3.16, 4.67)
−**2 to<**−**1**	287	1.52 (1.28, 1.81)	286	1.72 (1.43, 2.08)
**≥**−**1**	254	Ref	251	Ref
**Height/Length-for-Age Z score**				
**<**−**3**	477	5.48 (4.62, 6.50)	381	6.58 (5.50, 7.87)
−**3 to<**−**2**	305	2.28 (1.91, 2.72)	318	2.78 (2.33, 3.30)
−**2 to<**−**1**	283	1.46 (1.23, 1.74)	328	1.62 (1.37, 1.91)
**≥**−**1**	239	Ref	277	Ref
**Weight-for-Length/Height Z score**				
**<** −**3**	220	11.63 (9.84, 13.76)	120	17.71 (14.28, 21.97)
−**3 to<**−**2**	205	3.38 (2.86, 3.98)	225	4.96 (4.17, 5.90)
−**2 to<**−**1**	308	1.62 (1.41, 1.87)	394	1.87 (1.62, 2.15)
**≥** −**1**	571	Ref	565	Ref

a Models were stratified on cohort and adjusted for age (as the time scale, in weeks), child’s sex and assigned treatment in randomized trials.

**Figure 1:**
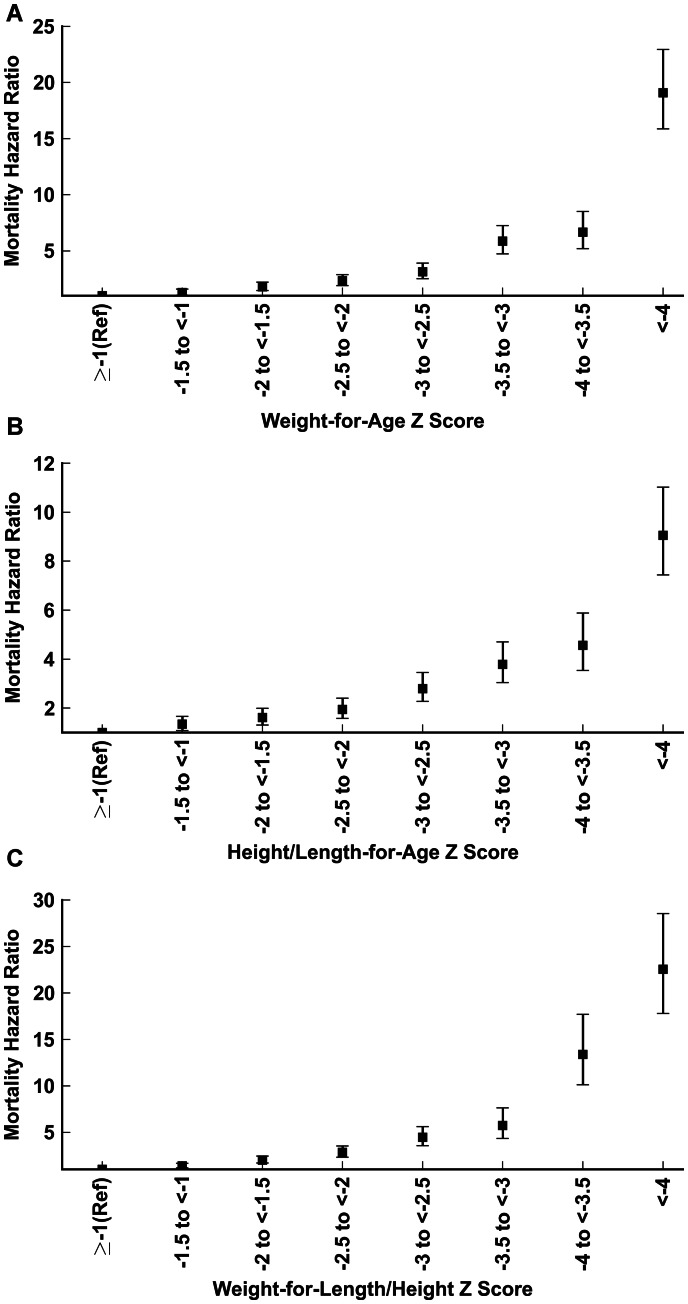
Associations of anthropometric measures (in increments of 0.5 Z scores) with all-cause mortality, WHO 2006 child growth standards. (A) Weight-for-age; (B) Height/length-for-age; (C) Weight-for-Height/Length.

Mortality HRs using decrements of 0.5 Z scores are presented in Figure 1, which shows a clear dose-response curve for all 3 anthropometric indices. Mortality hazards were elevated by as much as 22 fold (17.80, 28.54) for very severely wasted children with WHZ<−4, and HRs reached 9.05 (7.44, 11.02) and 19.09 (15.88, 22.94) for very severely stunted and underweight children, respectively.

All-cause mortality HRs were larger when the NCHS/WHO 1977 reference was used compared with HRs estimated using the WHO 2006 standards. WAZ hazard ratios for all-cause mortality were 13% to 46% larger with the NCHS/WHO 1977 reference. HAZ hazard ratios were 11% to 22% larger, and WHZ hazard ratios were 15% to 52% larger (Table 3).

Table 4 shows HRs from minimally and maximally adjusted models in 6 cohorts that had data on some additional potential confounders. The pooled point estimates of the maximally adjusted HRs were generally lower than the minimally adjusted HRs (by 3–10% for WAZ, 8–14% for HAZ and 0.8–6% for WHZ). However, the 95% confidence intervals for these estimates overlapped substantially with those of the minimally adjusted models, suggesting that the magnitude of confounding by the measured variables in these cohorts was small.

**Table 4:** Minimally-adjusted^a^ versus maximally-adjusted hazard ratios (HR) for 6 cohorts^b^ that reported additional baseline covariates beyond sex and randomized treatment assignment, WHO 2006 standards.

	Minimally adjusted hazard ratios			Maximally adjusted hazard ratios		
	Pooled^c^ HR (95% CI)	I^2^	p value^d^	Pooled^c^ HR (95% CI)	I^2^	p value^d^
**Weight-for-Age Z score**						
**<** −**3**	12.80 (6.97, 23.49)	42%	0.12	11.88 (6.03, 23.43)	49%	0.08
−**3 to<**−**2**	3.39 (2.12, 5.41)	0%	0.57	3.06 (1.79, 5.22)	15%	0.32
−**2 to<**−**1**	1.72 (1.08, 2.73)	0%	0.9	1.67 (1.04, 2.70)	0%	0.85
**≥** −**1**	Ref			Ref		
**Height/Length-for-Age Z score**						
**<** −**3**	6.41 (3.77, 10.89)	29%	0.21	5.50 (3.04, 9.98)	39%	0.14
−**3 to<**−**2**	2.45 (1.56, 3.87)	0%	0.61	2.12 (1.33, 3.38)	0%	0.63
−**2 to<**−**1**	1.56 (0.98, 2.46)	0%	0.97	1.44 (0.90, 2.30)	0%	0.99
**≥** −**1**	Ref			Ref		
**Weight-for-Length/Height Z score**						
**<** −**3**	14.32 (8.76, 23.41)	28%	0.22	14.20 (7.98, 25.27)	42%	0.13
−**3 to<**−**2**	3.70 (2.43, 5.61)	0%	0.78	3.47 (2.24, 5.36)	0%	0.84
−**2 to<**−**1**	1.67 (1.13, 2.46)	0%	0.95	1.61 (1.08, 2.42)	0%	0.81
**≥** −**1**	Ref			Ref		

a Adjusted for age (as the time scale, in weeks), child’s sex, cohort characteristics, and assigned treatment (in randomized trials).

b Nepal, Indonesia, Philippines, Bangladesh, Sudan, Guinea-Bissau.

c Hazard ratios were estimated in each cohort separately and then pooled using a random effects meta-analysis (see Methods).

d p value for test of no heterogeneity.

### Effects on Cause-specific Mortality

Of the 1315 deaths among eligible children, 371 were from diarrheal diseases, 187 from respiratory tract infections, 51 from malaria, 80 from measles, and 90 from ‘other infectious causes’. Having a mild anthropometric deficit (i.e. −2≤Z<−1) was associated with significantly higher mortality HRs ranging from 1.55 to 1.92 across different anthropometric indices for respiratory tract infections and from 1.60 to 1.73 for diarrheal diseases (Table 5). Moderate deficits (i.e. −3≤Z<−2) also increased the hazard of dying from measles with HRs ranging from 2.58 to 3.12. Severe anthropometric deficits (Z<−3) increased the hazard of dying from ‘other infectious causes’ (HRs ranging from 3.01 to 11.21) (Table 5). While the HRs for mild categories were not statistically significant for measles and other infectious diseases, there was a suggestion of a dose-response relationship. The largest effects were seen for deaths from diarrheal diseases, with HRs of 12.33 (9.18, 16.57) for severe wasting, and 11.56 (8.63, 15.48) for severe underweight. Three cohorts reported malaria deaths, but the number of deaths was not large enough to provide precise estimates of the effects of undernutrition.

**Table 5:** Minimally adjusted^a^ hazard ratio (HR) estimates for specific causes of mortality, WHO 2006 standards.

	Mortality from respiratory tract infections		Mortality from diarrheal disease		Mortality from other infectious causes^b^		Mortality from malaria^c^		Mortality from measles^d^	
	No. ofdeaths	HR (95% CI)	No. ofdeaths	HR (95% CI)	No. ofdeaths	HR (95% CI)	No. ofdeaths	HR (95% CI)	No. ofdeaths	HR (95% CI)
**Weight-for-Age Z score**										
**<** −**3**	68	10.10 (6.53, 15.64)	154	11.56 (8.63, 15.48)	36	8.28 (4.32, 15.89)	3	1.29 (0.39, 4.29)	26	7.73 (4.15, 14.39)
−**3 to<**−**2**	41	3.11 (1.93, 5.02)	74	2.86 (2.03, 4.03)	13	1.58 (0.73, 3.45)	10	1.65 (0.77, 3.53)	23	3.12 (1.67, 5.80)
−**2 to<**−**1**	43	1.85 (1.17, 2.91)	77	1.73 (1.24, 2.40)	21	1.54 (0.78, 3.03)	16	1.26 (0.66, 2.39)	13	1.00 (0.49, 2.03)
**≥** −**1**	35	Ref	66	Ref	19	Ref	22	Ref	18	Ref
**Height/Length-for-Age Z score**										
**<** −**3**	61	6.39 (4.19, 9.75)	136	6.33 (4.64, 8.65)	29	3.01 (1.55, 5.82)	10	1.92 (0.89, 4.11)	29	6.01 (3.00, 12.07)
−**3 to<**−**2**	38	2.18 (1.39, 3.43)	79	2.38 (1.71, 3.31)	24	1.86 (0.97, 3.57)	11	1.06 (0.48, 2.32)	22	2.79 (1.40, 5.56)
−**2 to<**−**1**	46	1.55 (1.02, 2.37)	85	1.67 (1.20, 2.30)	17	0.95 (0.48, 1.87)	12	0.74 (0.35, 1.56)	15	1.25 (0.61, 2.58)
**≥** −**1**	41	Ref	66	Ref	20	Ref	18	Ref	14	Ref
**Weight-for-Length/Height Z score**										
**<** −**3**	28	9.68 (6.07, 15.43)	73	12.33 (9.18, 16.57)	14	11.21(5.91, 21.27)	1	1.24 (0.17, 9.29)	13	9.63 (5.15, 18.01)
−**3 to<**−**2**	41	4.66 (3.07, 7.09)	61	3.41 (2.52, 4.63)	11	2.73 (1.35, 5.54)	4	1.43 (0.52, 3.94)	12	2.58 (1.32, 5.06)
−**2 to<**−**1**	47	1.92 (1.31, 2.84)	83	1.60 (1.23, 2.11)	23	1.65 (0.98, 2.79)	8	0.86 (0.39, 1.90)	15	1.02 (0.56, 1.85)
**≥** −**1**	70	Ref	149	Ref	42	Ref	38	Ref	40	Ref

a Adjusted for age (as the time scale, in weeks), child’s sex, cohort characteristics, and assigned treatment (in randomized trials).

b Septicemia, unspecified febrile illness, tuberculosis, meningitis, hepatitis or cellulitis.

c hree cohorts reported malaria deaths [23], [27], [29].

d even cohorts reported measles deaths [23]–[27], [29], [30].

### Sensitivity Analyses

Sensitivity analyses showed that our results were robust to the duration of time used to carry forward the last measurements of anthropometric indices (see Table S3). Similarly, results were robust to the assignment of 39 deaths to one or the other of the two reported causes of death. HRs changed by less than 14% in the latter sensitivity analyses, and the differences were not statistically significant.

## Discussion

Although it has been known that undernutrition increases mortality in children, effect estimates from the 2008 Lancet Series re-analyses using the new WHO growth standards noticeably differed from results of the CRA analysis which used the NCHS/WHO 1977 reference, especially for cause-specific mortality. Using pooled data from 10 prospective cohorts, we found that childhood undernutrition is strongly associated with elevated all-cause mortality when either the currently recommended WHO 2006 child growth standards or the older NCHS/WHO 1977 reference were used. The associations remained significant after adjusting for additional confounders in 6 cohorts. We also observed a clear dose-response relationship between the three anthropometric indices and under-five mortality. Using finer categories revealed a smooth dose-response relationship with a 20 fold increased mortality hazard for very severely wasted children. Furthermore, undernutrition was found to increase the rate of dying from infections including those of the gastrointestinal and respiratory tracts.

Given the relatively small number of malaria deaths, we had less than 45% power to detect the observed HR estimates. Therefore, we were unable to make any statement about childhood undernutrition and malaria mortality, which would require a larger study in populations where malaria is a common cause of death. Some prior studies reported an increased risk of malaria morbidity and mortality as a result of undernutrition. These studies included clinic or hospital-based studies [34]–[38] and cross-sectional studies [39], [40] which did not distinguish between preexisting and recent undernutrition (a plausible consequence of the acute malaria illness which caused hospitalization and eventual death [41]). Some reports have also suggested protective effects of undernutrition on severe malaria. Some of these studies involved study populations that comprised entirely of cerebral malaria patients or deaths due to malaria [42], [43], others lacked an adequately nourished comparison group [44]–[46], and none adjusted for confounding by socioeconomic and other factors which may have been responsible for the observed association. Children from lower socioeconomic backgrounds may be more frequently exposed to mosquito bites and malaria parasitemia and therefore develop some immunity and protection against severe manifestations [47]. Such children are also more likely to be undernourished, leading to the paradoxical finding of lower malaria mortality in undernourished compared to adequately nourished children. A few other prospective cohort studies in malaria-endemic populations did not find a significant association between childhood undernutrition and malaria morbidity [48]–[50].

The results of the current analysis are consistent with a previous pooling analysis in 2008 [1] though the magnitudes of association from our analysis are larger. The odds ratios in the previous pooling study were obtained using a logistic regression model and after applying a 15% attenuation to adjust for confounding by socioeconomic factors, determined from two studies in Nepal and Honduras [1]. We used a proportional hazard model to incorporate censoring due to loss to follow-up and adjusted for confounding in each study separately because relationships between potential confounders and exposures or outcome may differ across cohorts. Another potential reason for the larger effect estimates reported here may be the use of updated anthropometric indices as opposed to baseline measures which were used in the previous study. Baseline measures are not prone to confounding by disease status during follow-up but may not reflect the nutritional status of the child close to the time of death, which is especially relevant for underweight and wasting that may have a short latency time for their effect on mortality.

Our results, based on the same cohort studies and identical analytical methods, resolve any questions about the role played by the change in growth standards in producing the differences in effect estimates reported by the CRA Study [16] and the 2008 Lancet Series [1]. The WHO 2006 standards tend to classify children in lower Z score categories than the NCHS/WHO 1977 reference and this reclassification leads to lower HRs in the deficient categories. However, the differences with HRs using the old reference were often not statistically significant.

A commonly accepted pathway through which poor growth may increase mortality risk is through secondary immune suppression and increased susceptibility to infections, with worsening undernutrition in a vicious cycle. Undernutrition impairs the immune response, specifically that of innate and cell-mediated immune response [2], [51]–[53]. Immunological studies have shown that severe undernutrition may decrease immunoglobulin A production [54], [55], and impair T lymphocyte function [56], [57] and cytokine production [58]. Undernourished individuals may also suffer from impaired inflammatory response due to reduced synthesis of modulating molecules such as Interlukine-1 and Interlukine-6 [58], reduction in chemokines needed for macrophage mobilization, and impaired phagocytosis and microbial killing due to reductions in complement protein C3 [2], [51]. Acting together, these impairments result in decreased resistance to infections. Other complications of undernutrition may include fluid and electrolyte imbalances, hypoglycemia, hypothermia, and cardiac and respiratory dysfunction [59], [60].

Strengths of our study include the use of individual-level data from 10 large cohort studies. We used the most recent measures of anthropometric status as opposed to baseline values because the updated measures reflect the more recent nutritional status which may be more relevant for acute outcomes. We used a standard definition of anthropometric status categories [1], [16], as well as finer categories to provide a better understanding of the dose-response relationship between undernutrition and mortality. Our analyses utilized the more up-to-date WHO standards of 2006 and provided a comparison with results when the NCHS/WHO 1977 reference is used.

Our results should be interpreted with some limitations in mind. As in any observational analysis, confounding by unmeasured factors is a possibility. While many potential confounders of interest were not available in all studies, six cohorts measured a large number of potential confounders including socioeconomic characteristics. When these factors were included in the maximally adjusted models, results remained unchanged. Another potential confounder is presence and severity of disease. Having an illness may alter a child’s nutritional status and may also increase mortality, leading to potential positive confounding. However, we could not adjust for disease status because this information was not available in most cohorts. Furthermore, specific causes of death were determined by verbal autopsy in most studies, and the possibility of misclassification cannot be excluded. We expect any such misclassification to be independent of exposure values and thus not introduce any bias in the reported hazard ratios. Finally, we may have observed larger effects for wasting compared with underweight or stunting because the true exposure window for underweight and stunting may be longer than the average duration between exposure and death in our study.

Our findings have important policy implications. While the anthropometric status of children in many developing countries has improved over the past three decades [4] and under-5 mortality has declined [61], some countries in sub-Saharan Africa and South Asia have not yet experienced adequate progress [4], [61], [62]. Recent estimates suggest that the current pace is inadequate to achieve the first Millennium Development Goal (MDG 1) of eradicating extreme poverty and hunger: developing countries as a group have less than a 5% chance of meeting the target, and the probability is less than 50% for more than half of the countries [4]. New initiatives such as the UN “Global Strategy for Women’s and Children’s Health” [19], [20] and the UNICEF’s “Committing to Child Survival: A Promise Renewed” [21] are attempting to refocus the attention of governments and partners on addressing health challenges of children worldwide. Findings from this study should motivate continued support for the implementation of interventions which have been shown to be effective, and encourage research that explores new interventions and delivery strategies. For instance, promoting exclusive breastfeeding, complementary feeding and the WHO’s ‘case management of severe acute malnutrition’ guidelines have been shown to improve survival and reduce stunting in children [63]. To further reduce child mortality globally, sustained commitment is required beyond the MDG target year of 2015.

## Supporting Information

Table S1 Additional baseline covariates adjusted for in maximally adjusted analyses.
(DOCX)

Table S2 Study-specific and pooled hazard ratios (HR) for all-cause mortality using WHO 2006 standards for (A) weight-for-age; (B) height/length-for-age; and (C) weight-for-height/length.
(DOCX)

Table S3 Mortality hazard ratios (HR) in several sensitivity analyses, WHO 2006 standards.
(DOCX)
