# Supplementary material for: Associations of Suboptimal Growth with All-Cause and Cause-Specific Mortality in Children under Five Years: A Pooled Analysis of Ten Prospective Studies
Source: PLoS One. 2013 May 29;8(5):e64636. doi: 10.1371/journal.pone.0064636 (PMC3667136; doi:10.1371/journal.pone.0064636)
Supplement: Table S1 — Additional baseline covariates adjusted for in maximally adjusted analyses. (DOCX) [file pone.0064636.s001.docx]

**Table S1.** Additional baseline covariates adjusted for in maximally adjusted analyses

| **Cohort Name** | **Covariates adjusted for** |
| --- | --- |
| Nepal | Household assets ^a^ (household ownership of: bari, khet; number of household owned: bicycles, cattle, goats, radios; materials used for house walls and roof), mother’s education, mother’s age, household latrine ownership, household caste |
| Sudan | Household assets ^a^ (household ownership of: car, radio, television, refrigerator), mother’s literacy status, mother’s age, family size, private latrine ownership, household ownership of internal water supply, region of the country |
| Bangladesh | Household assets index, monthly household income, mother’s education, mother’s age, household religion, number of mother’s previous live births |
| Indonesia | Mother’s education, mother’s age, household drinking water source, family size |
| Philippines | Sanitation variables (i.e., animals kept under the house, domestic animals kept inside the house, condition of food cooking area, condition of food storage area), whether or not the child’s mother works for pay, source of baby’s drinking water, household water supply ^a^ (availability of: boreholes, dug wells, piped supply, springs) |
| Guinea-Bissau | Ethnic group |

^a^ Principal components analysis was used for data reduction of available variables and the first principal component was included in the multivariate model where applicable.
