# Supplementary material for: Associations of Suboptimal Growth with All-Cause and Cause-Specific Mortality in Children under Five Years: A Pooled Analysis of Ten Prospective Studies
Source: PLoS One. 2013 May 29;8(5):e64636. doi: 10.1371/journal.pone.0064636 (PMC3667136; doi:10.1371/journal.pone.0064636)
Supplement: Table S2 — Study-specific and pooled hazard ratios (HR) for all-cause mortality using WHO 2006 standards for (A) weight-for-age; (B) height/length-for-age; and (C) weight-for-height/length. (DOCX) [file pone.0064636.s002.docx]

**Table S2.** Study-specific and pooled hazard ratios (HR) for all-cause mortality using WHO 2006 standards for (A) weight-for-age; (B) height/length-for-age; and (C) weight-for-height/length

**(A)**

|  | **Hazard Ratio ^a^ (95% confidence interval)** | | |
| --- | --- | --- | --- |
|  | **Weight-for-Age Z (WAZ) score ^b^** | | |
| **Study** | **< - 1 to -2** | **< -2 to -3** | **< - 3** |
| **Bangladesh** | 2.64 (1.08, 6.43) | 4.15 (1.69, 10.17) | 21.57 (9.34, 49.78) |
| **Ghana** | 1.13 (0.60, 2.14) | 1.74 (0.80, 3.77) | 4.21 (2.02, 8.76) |
| **Guinea Bissau** | 1.22 (0.75, 1.97) | 2.28 (1.31, 3.96) | 6.08 (3.57, 10.36) |
| **India** | 2.03 (0.73, 5.64) | 2.94 (1.06, 8.16) | 19.93 (7.98, 49.78) |
| **Indonesia** | 1.31 (0.80, 2.13) | 2.53 (1.55, 4.13) | 6.40 (3.94, 10.42) |
| **Nepal** | 2.65 (0.90, 7.79) | 5.15 (1.83, 14.54) | 19.89 (7.28, 54.33) |
| **Peru** | 3.76 (1.01, 13.98) | 8.81 (1.71, 45.32) | 28.06 (4.91, 160.19) |
| **Philippines** | 2.11 (0.92, 4.84) | 8.56 (3.97, 18.46) | 39.18 (19.16, 80.11) |
| **Senegal** | 1.59 (1.21, 2.09) | 2.37 (1.77, 3.17) | 5.60 (4.15, 7.56) |
| **Sudan** | 1.82 (1.01, 3.27) | 2.50 (1.38, 4.53) | 10.57 (6.23, 17.93) |
| **Pooled HR^c^** | **1.69 (1.19, 2.40)** | **3.04 (2.12, 4.36)** | **10.96 (6.80, 17.66)** |
| **p for pooled HR** | **0.003** | **<0.0001** | **<0.0001** |
| **p^d^** | 0.96 | 0.68 | 0.08 |
| **I^2^** | 0% | 0% | 42.13% |

**(B)**

|  | **Hazard Ratio ^a^ (95% confidence interval)** | | |
| --- | --- | --- | --- |
|  | **Height/Length-for-Age Z (HAZ) score ^b^** | | |
| **Study** | **< - 1 to -2** | **< -2 to -3** | **< - 3** |
| **Bangladesh** | 1.85 (1.04, 3.29) | 3.44 (1.90, 6.22) | 12.15 (7.03, 20.97) |
| **Ghana** | 0.89 (0.47, 1.71) | 1.16 (0.55, 2.46) | 4.36 (2.19, 8.65) |
| **Guinea Bissau** | 1.35 (0.79, 2.31) | 1.79 (1.03, 3.13) | 3.61 (2.07, 6.29) |
| **India** | 1.59 (0.74, 3.41) | 3.52 (1.70, 7.28) | 8.83 (4.37, 17.88) |
| **Indonesia** | 1.32 (0.67, 2.59) | 1.84 (0.97, 3.48) | 4.12 (2.24, 7.60) |
| **Nepal** | 1.33 (0.95, 1.87) | 1.70 (0.70, 4.15) | 9.34 (4.21, 20.70) |
| **Peru** | 2.90 (0.86, 9.85) | 9.44 (2.57, 34.64) | 12.09 (1.47, 99.25) |
| **Philippines** | 2.37 (1.10, 5.09) | 5.59 (2.71, 11.53) | 14.98 (7.25, 30.92) |
| **Senegal** | 1.44 (1.09, 1.90) | 1.98 (1.47, 2.67) | 3.70 (2.75, 4.97) |
| **Sudan** | 1.25 (0.70, 2.21) | 1.63 (0.94, 2.84) | 3.61 (2.19, 5.95) |
| **Pooled HR^c^** | **1.51 (1.07, 2.12)** | **2.44 (1.73, 3.46)** | **5.81 (4.07, 8.30)** |
| **p for pooled HR** | **0.02** | **<0.0001** | **<0.0001** |
| **p^d^** | 0.98 | 0.5 | 0.39 |
| **I^2^** | 0% | 0% | 6% |

**(C)**

|  | **Hazard Ratio ^a^ (95% confidence interval)** | | |
| --- | --- | --- | --- |
|  | **Weight-for-Height/Length Z (WHZ) score ^b^** | | |
| **Study** | **< - 1 to -2** | **< -2 to -3** | **< - 3** |
| **Bangladesh** | 1.49 (0.90, 2.47) | 3.22 (1.92, 5.38) | 9.36 (5.65, 15.53) |
| **Ghana** | 1.46 (0.71, 3.02) | 2.94 (1.14, 7.56) | ---^e^ |
| **Guinea Bissau** | 1.51 (0.92, 2.46) | 2.37 (1.18, 4.77) | 5.85 (2.71, 12.66) |
| **India** | 1.86 (0.98, 3.54) | 4.68 (2.44, 8.99) | 18.94 (10.41, 34.46) |
| **Indonesia** | 2.21 (1.54, 3.16) | 3.40 (1.97, 5.84) | 10.00 (5.44, 18.39) |
| **Nepal** | 1.55 (0.96, 2.51) | 4.96 (3.14, 7.84) | 14.84 (9.19, 23.95) |
| **Peru** | 1.47 (0.18, 11.77) | 13.02 (1.83, 92.75) | ---^e^ |
| **Philippines** | 2.14 (1.25, 3.65) | 6.78 (3.89, 11.80) | 39.00 (23.52, 64.66) |
| **Senegal** | 1.61 (1.25, 2.07) | 2.50 (1.81, 3.46) | 5.41 (3.65, 8.01) |
| **Sudan** | 1.26 (0.83, 1.94) | 2.87 (1.75, 4.72) | 19.10 (12.77, 28.58) |
| **Pooled HR^c^** | **1.65 (1.21, 2.25)** | **3.64 (2.60, 5.09)** | **12.72 (7.98, 20.28)** |
| **p for pooled HR** | **0.002** | **<0.0001** | **<0.0001** |
| **p^d^** | 0.99 | 0.8 | 0.1 |
| **I^2^** | 0% | 0% | 41.75% |

^a^ Hazard Ratios (HR) and 95% confidence intervals (CI) from Cox proportional hazards regression models with age in weeks as time scale, adjusting for sex, and assigned treatment (in randomized trials)

^b^ Reference group was Z score ≥ - 1

^c^ Pooled HR from random effects meta-analysis using the DerSimonian and Laird method

^d^ p value for test of no heterogeneity

^e^ No events occurred in the WHZ <-3 category for the Ghana and Peru studies; hazard ratios could not be estimated
