# Supplementary material for: Associations of Suboptimal Growth with All-Cause and Cause-Specific Mortality in Children under Five Years: A Pooled Analysis of Ten Prospective Studies
Source: PLoS One. 2013 May 29;8(5):e64636. doi: 10.1371/journal.pone.0064636 (PMC3667136; doi:10.1371/journal.pone.0064636)
Supplement: Table S3 — Mortality hazard ratios (HR) in several sensitivity analyses, WHO 2006 standards. (DOCX) [file pone.0064636.s003.docx]

**Table S3.** Mortality hazard ratios (HR) in several sensitivity analyses, WHO 2006 standards

|  | **Z scores carried forward no more than 4 months** | **Definition of causes: respiratory tract infections ^a^** | **Definition of causes: diarrheal diseases ^a^** |
| --- | --- | --- | --- |
|  | **HR (95% CI)** | **HR (95% CI)** | **HR (95% CI)** |
| **Weight-for-Age Z score** |  |  |  |
| **< -3** | 10.55(8.79, 12.67) | 11.06(7.25, 16.86) | 11.99(8.99, 15.99) |
| **-3 to < -2** | 2.84 (2.31, 3.48) | 3.18 (1.99, 5.08) | 2.93 (2.09, 4.10) |
| **-2 to < -1** | 1.62 (1.33, 1.98) | 1.87 (1.19, 2.93) | 1.77 (1.28, 2.45) |
| **≥ -1** | Ref | Ref | Ref |
| **Height/Length-for-Age Z score** |  |  |  |
| **< -3** | 6.00 (4.96, 7.26) | 6.90 (4.61, 10.32) | 6.60 (4.87, 8.95) |
| **-3 to < -2** | 2.45 (2.01, 2.98) | 2.26 (1.47, 3.49) | 2.42 (1.75, 3.34) |
| **-2 to < -1** | 1.49 (1.22, 1.81) | 1.50 (0.99, 2.27) | 1.64 (1.19, 2.25) |
| **≥ -1** | Ref | Ref | Ref |
| **Weight-for-Length/Height Z score** |  |  |  |
| **< -3** | 12.94(10.79, 15.52) | 11.17(7.28, 17.16) | 12.82(9.66, 17.03) |
| **-3 to < -2** | 3.52 (2.93, 4.23) | 5.02 (3.37, 7.48) | 3.51 (2.62, 4.70) |
| **-2 to < -1** | 1.69 (1.44, 1.98) | 1.96 (1.34, 2.86) | 1.56 (1.19, 2.04) |
| **≥ -1** | Ref | Ref | Ref |

^a^ 39 deaths in two studies overlapped respiratory tract infections and diarrheal disease cause of death categories. We examined sensitivity of results to their assignment to one or the other cause category
